# Cerebral phaeohyphomycosis in liver transplant recipient: A case report

**DOI:** 10.1002/ccr3.6691

**Published:** 2022-12-05

**Authors:** Kaleem Ullah, Muhammad Asif Baig, Abdul Wahab Dogar, Shams Uddin, Chaudhary Abdul Fatir, Ali Asad, Muhammad Junaid Tahir, Ka Yiu Lee, Khabab Abbasher Hussien Mohamed Ahmed, Zohaib Yousaf

**Affiliations:** ^1^ Pir Abdul Qadir Shah Jeelani Institute of Medical Sciences Gambat Pakistan; ^2^ Lahore General Hospital Lahore Pakistan; ^3^ Services Hospital Lahore Pakistan; ^4^ Department of Health Sciences Mid Sweden University Ostersund Sweden; ^5^ Faculty of Medicine University of Khartoum Khartoum Sudan; ^6^ Tower Health Reading Pennsylvania USA

**Keywords:** brain, fungal infection, immunocompromised, pathogens, *Rhinocladiella mackenziei*

## Abstract

Cerebral phaeohyphomycosis is a fungal brain infection with a high fatality rate. It is caused by dematiaceous fungi and is increasingly recognized as a cause of serious illness in both immunocompetent and immunocompromised patients. We report cerebral phaeohyphomycosis in a liver transplant recipient. He was treated with multiple surgeries and antifungals and made a complete recovery. This report highlights that early and aggressive surgical intervention and extended antifungal coverage can have a positive outcome even in immunocompromised patients. The fungal infection in immunocompromised patients should be considered and treated aggressively.

## Abstract 2

Cerebral phaeohyphomycosis is a fungal brain infection with a high fatality rate. Early and aggressive surgical intervention and extended antifungal coverage can have a positive outcome even in immunocompromised patients. The fungal infection in immunocompromised patients should be considered and treated aggressively.

## 1 INTRODUCTION

Cerebral phaeohyphomycosis is a deadly fungal brain infection. Common pathogens associated with this infection belong to dematiaceous fungi. Dematiaceous fungi are a heterogeneous group of ubiquitous fungi that form dark colonies and have pigmented hyphae.^1^ *Rhinocladiella mackenziei*, a neurotropic dematiaceous fungus of the group order Chaetothyriales, is an emerging cause of cerebral phaeohyphomycosis, especially in immunocompromised patients.^2^, ^3^ Naim et al. described this infection in the Middle East.^4^ Later, sporadic cases were also observed in other regions beyond the Middle East.^5^, ^6^, ^7^, ^8^ Cerebral phaeohyphomycosis has a high mortality. Despite aggressive treatment, the mortality rate approaches up to 100 percent in both immunocompetent and immunocompromised.^7^, ^9^ Due to the indolent nature of the disease, most cases are diagnosed incidentally or during autopsy.^10^

We present a case of cerebral phaeohyphomycosis secondary to *Rhinocladiella mackenziei* in an immunocompromised liver transplant recipient. After aggressive surgeries and antifungals, the patient made a complete neurological recovery.

## 2 CASE PRESENTATION

A 57‐year‐old gentleman, previously fit and well, a farmer by profession, underwent a living donor liver transplantation for decompensated chronic liver disease secondary to hepatitis B and D co‐infection. Post‐operatively, immunosuppressive therapy was started with oral prednisolone 20 mg once daily (tapered till the end of the third month), along with oral tacrolimus (with an adjusted dose for achieving a level of 5–8 ng/mL). He also received oral trimethoprim‐sulphamethoxazole and oral fluconazole 200 mg once daily for 3 months as prophylaxis for opportunistic infections as per local institutional protocol. His post‐transplant hospital course was uneventful.

At four‐month post‐transplantation, he presented with acute onset of non‐remitting painless blurred vision in both eyes for 2 weeks. This was accompanied by a one‐week history of continuous high‐grade fever, which was relieved temporarily with antipyretics. He also complained of a continuous, diffuse, and moderate dull headache for 1 week which did not relieve with oral analgesia. He also reported a week of non‐projectile, non‐bilious vomiting four to five times a day. He had no history of unconsciousness, seizures, vertigo, diplopia, and weakness.

On physical examination, his pulse was 78/minute, blood pressure was 124/82 mm of Hg, respiratory rate was 18/minute, and body temperature was 101 F. His Glasgow coma scale was 15/15. Nuchal rigidity, jolt accentuation, Kernig's sign, and Brudzinski's sign were absent. Cranial nerve examination and fundal examination were unremarkable. The patient had normal bulk, tone, reflexes, and power bilaterally in the upper and lower limbs. The sensory and cerebellar examination was unremarkable.

**FIGURE 1:**
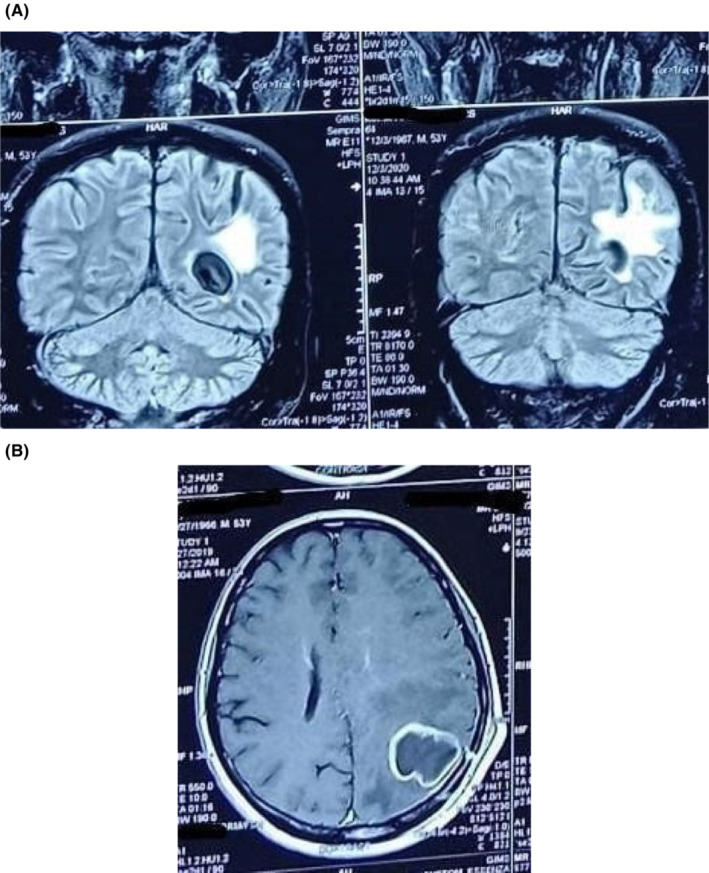
(A and B): MRI showing ring‐enhancing lesion with peripheral edema in the left occipital region

His complete blood count showed neutrophilic leukocytosis (12.4 × 10^9^/L, 85.3% neutrophils) and a raised C‐reactive protein (12.1 mg/L). Electrolytes, liver, and renal function tests were normal. Contrast‐enhanced T2‐weighted magnetic resonance imaging (MRI) brain showed a hyperintense single ring‐enhancing parieto‐occipital lesion 3.4 cm × 2.5 cm × 3.9 cm with peripheral vasogenic edema suggestive of a brain abscess. (Figure 1).

**FIGURE 2:**
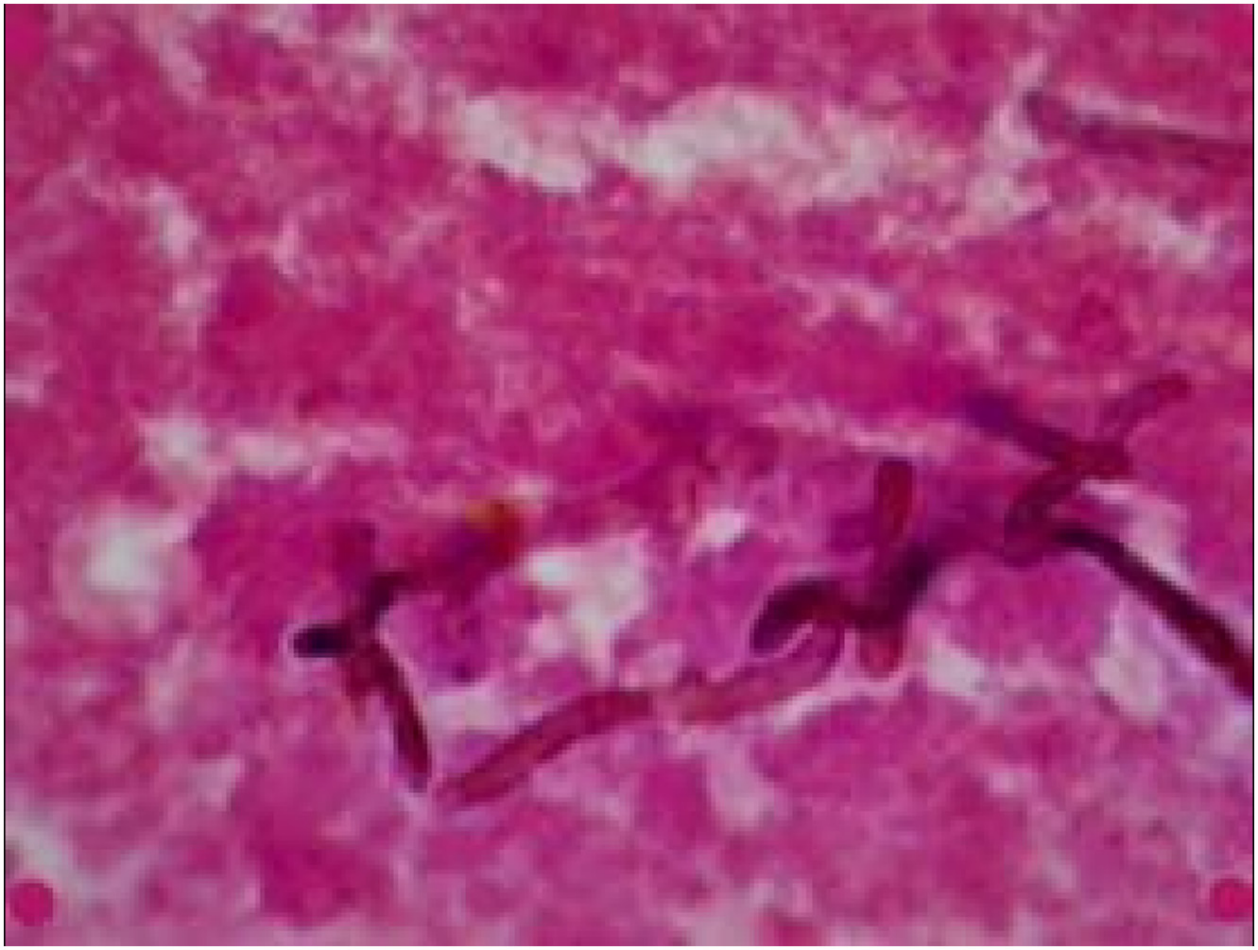
Gram stain and hematoxylin and eosin (H&E) stain showing fungal septate hyphae with a diffuse mixed inflammatory infiltrate (10× and 40× magnification)

He was admitted and empirically started on intravenous meropenem 2 gm eight hourly and intravenous vancomycin 500 mg six hourly with a working diagnosis of brain abscess. The surgical intervention was decided by Multidisciplinary team including the neurosurgery team, infectious disease team, and the internal medicine team based on MRI brain findings. He underwent a parieto‐occipital craniotomy for excision of the lesion along with drainage of about 120 ml of thick creamy yellow pus. The tissue sample was mixed with 10% KOH and visualized at 10× and 40× magnification. It was initially stained with hematoxylin and eosin stains which showed inflammatory exudate with numerous moderate septate hyphae (Figure 2). Further staining with periodic acid‐Schiff highlighted the presence of moderate to darkly pigmented fungal septate hyphae. The patient was started on intravenous fluconazole 400 mg once daily based on suspicion of fungal infection.

Specimens were inoculated on Saboraud's dextrose agar (Merck, Germany), sheep blood agar (Merck, Germany), and potato dextrose agar (Merck, Germany). The plates were incubated at 25 and 37°C and were observed daily. After 3 weeks of inoculation, the growth of greenish colonies with raised peripheral edges was seen. Then, slide culture was prepared on malt extract agar (Merck, Germany) and mature sporulating colonies of *Rhinocladiella mackenziei* at 6 weeks of incubation, that is, Mickey Mouse appearance was finally seen. Since the facility of molecular testing for this rare fungi was not available at our institute, so, the diagnosis was made only based on morphological characteristics. Also, the antifungal sensitivity testing was not done due to the unavailability of the facility. The patient was switched to intravenous (IV) voriconazole (200 mg) twice daily on the recommendation of the infectious disease(ID) team after getting the culture report.

However, the patient's symptoms, including headache and fever, did not subside 2 weeks after the surgery. On fifteen post‐operative day, MRI brain was repeated which showed residual brain abscess. So re‐evacuation was planned for source control. The residual brain abscess was drained, and the pus was re‐cultured. Repeated cultures grew the same organism.

One week after the 2nd surgery, the patient's fever resolved completely. He was discharged after 1 month of total hospital stay. The headache also resolved after 1 month. The patient recovered completely without developing any focal neurological deficit. At discharge, he was advised voriconazole 200 mg once daily orally for 1 year by the ID team. Long‐term voriconazole therapy was discussed with the patient. Patient compliance was good and he completed therapy for 1 year.

During this illness, the patient was continued on low‐dose tacrolimus (0.5 mg twice daily) due to ongoing sepsis. However, 1 month after his recovery, the tacrolimus dose was adjusted to achieve blood tacrolimus levels of 5–8 ng/mL. His liver function test remained stable throughout the illness despite being on a lower immunosuppression dose. The patient is doing well until the last follow‐up (one‐year post‐surgery).

## 3 DISCUSSION

*Rhinocladiella mackenziei* is being reported increasingly with cerebral phaeohyphomycosis.^11^ A literature review conducted on November 9, 2022, on PubMed using the Boolean operator strategy of “(Rhinocladiella mackenziei) AND (Cerebral phaeohyphomycosis)” revealed 14 articles with 12 relevant articles. Most cases were reported from the Middle East and Southeast Asia.^11^, ^12^ However, a few sporadic cases were also reported from North America and Europe.^8^, ^13^ Few similar cases were found in literature from Pakistan. However, all the reported patients were immunocompetent.^9^

Rhinocladiella mackenziei can cause respiratory infections, deadly brain abscesses, and widespread sepsis.^14^, ^15^ Cerebral phaeohyphomycosis clinically presents as fever, headaches, behavioral changes, seizures, and focal neurological deficit, depending on the lesion's size, number, location, and duration.^15^, ^16^

The hallmark of cerebral phaeohyphomycosis is the presence of a brain abscess. Immunodeficiency like solid organ transplantation, chronic liver disease, connective tissue disorders, and chronic steroid use can predispose to cerebral phaeohyphomycosis.^14^ There were no identifiable underlying risk factors in multiple patients with cerebral phaeohyphomycosis. Our patient was immunodeficient considering the immunosuppressive therapy but was successfully treated with extensive surgery and antifungal coverage.

Literature review reported a single brain abscess in 51.6% of patients while multiple brain abscesses in 48.4% of the patients.^12^ Usually, immunocompetent individuals present with a single abscess, while immunocompromised can have multiple abscesses. The lesion on MRI shows typical ring enhancement on T1‐weighted images and hyperintensity on T2 images.^15^, ^17^ In this patient, similar MRI findings were appreciated.

The most recent joint European Confederation of Medical Mycology (ECMM) and European Society of Clinical Microbiology and Infectious Diseases (ESCMID) clinical guidelines for cerebral phaeohyphomycosis treatment recommend complete control of the source of infection along with antifungal coverage.^12^, ^18^ Our patient was also treated according to these recommendations.

Despite treatment, cerebral phaeohyphomycosis has a high mortality. The average survival is up to 4.7 months.^12^ The commonly used antifungal drugs are voriconazole, amphotericin B, itraconazole, and posaconazole. Single and combination therapies have been tried.^15^ Literature also reports mixed results regarding the efficacy of various antifungals drugs in cerebral phaeohyphomycosis.^12^, ^15^ Yusupov et al. and others recommended posaconazole for cerebral phaeohyphomycosis secondary to *Rhinocladiella mackenziei*.^15^, ^16^, ^19^, ^20^ Mohammadi, et al. recommended voriconazole over posaconazole.^12^ The updated joint ECMM /ESCMID guidelines also favor voriconazole, which is more efficient at penetrating the meninges. Our patient received voriconazole for 1 year.

## 4 CONCLUSIONS

Cerebral phaeohyphomycosis caused by *Rhinocladiella mackenziei* is a life‐threatening brain infection with high morbidity and mortality. Early and extensive surgical intervention with extended antifungal coverage can have a favorable outcome. Cerebral phaeohyphomycosis should be considered in immunocompromised patients with central nervous system symptoms.

## AUTHOR CONTRIBUTIONS

**Kaleem Ullah:** Formal analysis; investigation; writing – original draft. **Muhammad Asif Baig:** Investigation; methodology; writing – review and editing. **Abdul Wahab Dogar:** Investigation; methodology; writing – review and editing. **Shams Uddin:** Investigation; methodology; writing – review and editing. **Chaudhary Abdul Fatir:** Investigation; methodology; writing – review and editing. **Ali Asad:** Investigation; methodology; writing – review and editing. **Muhammad Junaid Tahir:** Investigation; methodology; writing – review and editing. **Ka Yiu Lee:** Investigation; methodology; writing – review and editing. **Khabab Abbasher Mohamed Ahmed:** Investigation; methodology; writing – review and editing. **Zohaib Yousaf:** Investigation; methodology; writing – review and editing.

## CONFLICT OF INTEREST

None.

## ETHICAL APPROVAL

Written informed consent was obtained from the patient to publish this report in accordance with the journal's patient consent policy.

## CONSENT

Written informed consent was obtained from the patient to publish this report in accordance with the journal's patient consent policy.

## Data Availability

Data are available upon reasonable request from the first author (Kaleem Ullah).
